# Associations between gender, school socioeconomic status, and cardiorespiratory fitness among elementary and middle school students

**DOI:** 10.1186/s12889-020-09571-y

**Published:** 2020-10-02

**Authors:** Timothy J. Walker, Derek W. Craig, Andjelka Pavlovic, Shelby Thiele, Harold W. Kohl

**Affiliations:** 1 Department of Health Promotion & Behavioral Sciences, Center for Health Promotion and Prevention Research, University of Texas Health Science Center at Houston School of Public Health, 7000 Fannin St, Houston, TX 77030 USA; 2 Division of Youth Education, The Cooper Institute, 12330 Preston Road, Dallas, TX USA; 3 Department of Epidemiology, Human Genetics and Environmental Sciences, The University of Texas Health Science Center at Houston School of Public Health, Austin Regional Campus, Austin, TX USA; 4 Department of Kinesiology and Health Education, The University of Texas at Austin, 1616 Guadalupe, Austin, TX 78701 USA

## Abstract

**Background:**

Schools play an important role in providing access to physical activity opportunities for children. There are common economic and gender disparities in physical activity and health-related fitness among children, which may inform a school’s programming needs. The purpose of this study is to gain a better understanding about gender, school-level socioeconomic status, and children’s cardiorespiratory fitness.

**Methods:**

This observational study used 2017–2018 school year data from schools in the Dallas Metropolitan area participating in the Healthy Zone School (HZS) program. Three data sources were integrated: 1) FitnessGram® data, 2) school-level data from the Texas Education Agency, and 3) HZS survey data. Being in the Healthy Fitness Zone (HFZ) for aerobic capacity was the dependent variable, and gender and the percentage of economically disadvantaged students (at the school-level) were key independent variables. Mixed-effects logistic regression models examined associations between dependent and independent variables. Final models were adjusted for age and type of aerobic test.

**Results:**

There were 67 schools and 15,052 students included in the analysis. When testing main effects, girls had lower odds for being in the HFZ for aerobic capacity than boys (OR = 0.54, CI = 0.47–0.62). Additionally, having a greater percentage of students who were economically disadvantaged was associated with lower odds for being in the HFZ for aerobic capacity (0.98, CI = 0.98–0.99). There was a significant interaction between gender and the percentage of economically disadvantaged students. Results indicated girls had even lower odds (than boys) for being in the HFZ in schools with 90% economically disadvantaged students (OR = 0.44, CI = 0.35–0.55) versus in schools with 15% economically disadvantage students (OR = 0.62, CI = 0.51–0.76).

**Conclusions:**

Results suggest girls in Healthy Zone Schools have lower odds to meet aerobic capacity fitness standards than boys. Additionally, boys and girls in schools serving a greater percentage of economically disadvantaged students have lower odds to meet aerobic capacity fitness standards. Last, girls have even lower odds of meeting HFZ standard when attending a school serving a greater percentage of economically disadvantaged students. Thus, schools need to provide more programs for girls targeting aerobic physical activity. This is especially important for schools serving a high percentage of low-income students.

## Introduction

There is an important public health need to increase physical activity among children and address health disparities related to physical inactivity. Only about 49% of boys and 36% of girls aged 6–11 years are meeting the Physical Activity Guidelines for Americans recommendations of 60 min of daily activity [1, 2]. Girls tend to be less physically active than boys both during and outside of the school day [3–6]. In addition, the difference between boys’ and girls’ activity levels continue as children transition to adolescence and become even less active [2, 3]. These gender differences in physical activity have both short- and long-term health implications, which need to be better understood in the context of key health promotion settings.

Ecological health models theorize that health behaviors are influenced across multiple levels including policy, community, institution, interpersonal, and intrapersonal [7, 8]. For example, at the institution-level, schools play an important role in supporting children’s physical activity. Schools are recommended to take a Whole-of-School approach by promoting physical activity before, during, and afterschool [9]. Schools are well-positioned to deliver physical activity approaches because of their existing infrastructure and broad reach. They also have the potential to address health disparities by providing access to physical activity opportunities regardless of students’ gender, age, race/ethnicity, or socioeconomic status (SES). Despite the promise, there are many implementation challenges in schools, which include a poor implementation climate, competing priorities, lack of space, and lack of professional development opportunities [10–12]. These challenges impact the reach and effectiveness of school-based physical activity approaches and may vary across schools to differentially impact segments of the student population.

Previous research using nationally representative data suggests that school-level characteristics are associated with the implementation of physical activity approaches (e.g. classroom activity breaks, active learning lessons, recess) [13, 14]. More specifically, schools serving a higher percentage of students eligible for free/reduced lunch were less likely to have a 20-min daily recess period [13] and less likely to offer classroom-based physical activity approaches [14]. Schools serving low-income students are likely choosing to provide additional classroom instruction time rather than providing daily recess opportunities [13], or struggle to implement classroom-based approaches because of funding challenges [14]. As a result, students at these schools are missing important opportunities for physical activity, which can negatively impact health and further drive health disparities related to SES and gender.

Multiple studies have found gender differences between boys and girls physical activity levels. However, less is reported about gender differences in health-related fitness assessments such as cardiorespiratory fitness. Cardiorespiratory fitness refers to the function of the heart, lungs, blood vessels, and the muscular system involved in movement [15], and is independently related to health and academic performance [9]. Because children’s cardiorespiratory fitness can be estimated using validated field methods, and is related to a healthier cardiovascular profile later in life [16], it serves as a good indicator of health-related fitness for children. In addition to fewer studies focusing on gender differences in cardiorespiratory fitness, there is a lack of research examining how school-level characteristics may contribute to these differences in fitness levels. Gaining a better understanding about the relations between gender differences, school-level characteristics such as SES, and students’ cardiorespiratory fitness can help inform physical activity programming needs in schools. The objectives of this study are to: 1) examine gender differences in cardiorespiratory fitness among elementary and middle school students; 2) assess the relation between school-level SES and students’ cardiorespiratory fitness levels, and 3) determine whether gender differences vary across different levels of school SES.

## Methods

### Study design and setting

This observational study used data from the 2017–2018 school year from schools in the Healthy Zone School (HZS) program (www.healthyzoneschool.com) [17]. The HZS program was developed to help schools create a sustainable health promoting environment that supports physical activity, healthy eating, and other health behaviors. Participating schools completed surveys in the spring of 2018 about physical activity-related programs and activities offered throughout the school year to their students. As a part of participation in the HZS program, the FitnessGram assessment was administered at all participating schools. This set of validated field tests, designed to assess health-related fitness, is the most popular testing battery currently used in schools [15]. Schools consented to share their collected FitnessGram data. For this study, HZS survey data, FitnessGram data, and publicly available descriptive data from the Texas Education Agency [18] were integrated to examine research questions. The Institutional Ethics Review Boards of the University of Texas Health Science Center at Houston and The Cooper Institute approved this study.

### Participants

The HZS program was available to elementary, middle, and high schools, including public, private, and charter schools. Schools in the Dallas, Texas (USA) metropolitan area were eligible to apply to the program and selected to participate based on their current health policies. The program was advertised to schools through social media, promotional materials, local presentations to districts’ health advisory committee meetings. The HZS program enrolls a new cohort of schools on an annual basis and schools commit to the program for 3 years. One representative (usually a physical education teacher) at each participating school completed a HZS survey (additional details listed below) and provided consent for the study. In the 2017–2018 school year, there were 74 participating schools serving about 49,000 students. Overall, this study used a convenience sample of schools participating in the HZS program. To be included in this study, schools must have been participating in the HZS program, had TEA data, completed the HZS survey, and completed FitnessGram testing in the 2017–2018 school year. Student-level data were included for students in grade 3 (or higher) and were excluded if they did not have a valid aerobic capacity test conducted in the spring term of 2018 (January–July 2018).

### Measures

Cardiorespiratory fitness was assessed for each student through an aerobic capacity test from the FitnessGram assessment. Physical education teachers at each school tested student’s aerobic capacity using one of three tests: the 20-m pacer, 15-m pacer, or a one-mile run and entered their results into the FitnessGram software provided to schools. The number of laps completed during the pacer tests was converted to comparable one-mile run times using a test equating procedure [19]. Aerobic capacity was then predicted using one-mile run time, age, gender, and BMI and reported as estimates of V0_2max_ (maximal oxygen uptake) [20]. V0_2max_ values were calculated by the FitnessGram software when physical education teachers entered test results (either number of pacer laps or one-mile run time). Meeting the Healthy Fitness Zone (HFZ) standard for aerobic capacity represents a cardiorespiratory fitness level that is associated with health benefits. The HFZ standards for all FitnessGram tests, including aerobic capacity, are organized by age and gender-specific cut points [15, 21]. Schools in the HZS program collected FitnessGram data in the winter/spring of 2018. Other variables included in the FitnessGram data were student’s gender, age, grade level, and type of aerobic capacity test completed.

School-level descriptive variables were also included in the analysis. These variables were obtained from the Texas Academic Performance Reports (TAPR) from the Texas Education Agency (TEA) website for the corresponding 2017–2018 school year [18]. The TAPR reports include the total number of students enrolled at a school, the type of school (e.g. elementary, middle, high school), the percentage of students in each racial/ethnic category, the percentage of English language learners (students whose primary language is other than English), and the percentage of students who are economically disadvantaged (which was used as a measure of school-level SES). Economically disadvantaged was defined as students eligible for free or reduced-price lunch or eligible for other public assistance [22].

Additional school-level data were collected from the HZS survey, which was distributed to a representative at each school. The HZS survey included questions about the school’s use of programs and activities during the 2017–2018 school year. Programs were defined as any initiative that consisted of a planned series of events that targeted students’ physical activity (e.g., in-class activity breaks, running clubs, NFL Play 60 Challenge). Activities were a single event promoting physical activity that did not require continued participation over time (e.g., field day, family fitness night, pedometer challenges). Count variables were created based on the number of respective programs and activities that were reportedly used. Participants also responded to questions rating the success (on a 1–10 scale) of each program or activity used. The success scores were averaged across the respective programs and activities used. The program and activity variables were considered for inclusion in models to account for the number and perceived success of physical activity opportunities provided to students.

### Statistical analysis

Descriptive statistics were examined for both individual and school-level variables. Three-level mixed-effects logistic regression models examined the association between dependent and independent variables. Level-one was student, level-two was class, and level-three was school. Bivariate random intercepts models were examined for each student-level variable (gender, age, grade, and type of aerobic capacity test). A random coefficient model (for gender) was also tested and compared to the random intercepts model given the coefficients for gender could be different by school. Select school-level variables (in addition to percentage of economically disadvantaged students) were also considered to be included in final models if they were theoretically meaningful and statistically significant (which was determined by a likelihood-ratio test). These variables included school type, school size, percentage of students in racial/ethnic categories, percentage of English language learners, HZS cohort, and the number and success of physical activity programs and activities. To examine the combined effect, an interaction term between gender and percentage of economically disadvantaged students was included in an additional model. All modeling was conducted using Stata 15.1.

## Results

The final analytic sample consisted of 15,052 students from 287 classes and 67 schools. Seven schools were excluded from the analysis (4 schools lacked TEA data because they were not public schools, 2 schools did not report FitnessGram data, and 1 school did not have survey data). From the participating schools, 6698 students were not included in the analysis. The most common reason was because the student was too young for the aerobic capacity test (4965). Current FitnessGram standards are available for 3rd graders and above. Other reasons included the student had missing data (83), an incomplete test (453), was exempted from the test (529), or had an invalid score (668). Just under half of the analytic sample was girls (48%), the average age was 10.7 years, and a majority of the sample was in grades 3–5 (72%) (Table 1).

**Table 1:** Individual-level Characteristics (*n* = 15,052)

Variable	Total Sample	Meeting HFZ Standards for Aerobic Capacity	OR (95% CI)
Gender (n, %)			
Boy (ref)	7827 (52.0%)	6128 (78.3%)	1.00
Girl	7225 (48.0%)	4826 (66.8%)	0.49 (0.45–0.53)**
Age in years (m, sd)	10.7 (1.2)	10.7 (1.1)	0.75 (0.71–0.79)**
Grade (n, %)			
3–5 (ref)	10,810 (71.8%)	7967 (73.7%)	1.00
6–9	4242 (28.2%)	2987 (70.4%)	0.75 (0.64–0.86)**
Aerobic Capacity Test			
20 Meter Pacer	11,500 (76.4%)	8503 (73.9%)	1.00
15 Meter Pacer	1542 (10.2%)	771 (50.0%)	0.23 (0.12–0.42)**
One-Mile-Run	2010 (13.3%)	1680 (83.5%)	1.96 (1.16–3.33)*

**, *p* < 0.001; *p < 0.05

Results from bivariate random intercepts models indicated significant trends for each respective student-level variable (Table 1). Notably, girls had lower odds for meeting HFZ standards for aerobic capacity compared to boys (OR = 0.49, 95% CI = 0.45–0.53). Older students (and students in higher grades) had lower odds (OR = 0.75, 95% CI = 0.71–0.79, OR = 0.75, 95% CI = 0.64–0.86, respectively) for meeting HFZ standards for aerobic capacity compared to younger students (and students in lower grades). There also appeared to be a potential effect for the type of test completed. More specifically, students who completed the 15-m pacer test had lower odds for meeting HFZ standards compared to students who completed the 20-m pacer test (OR = 0.23, 95% CI = 0.12–0.42). Additionally, students who completed the one-mile-run test had higher odds for meeting HFZ standards compared to students who completed the 20-m pacer test (OR = 1.96, 95% CI = 1.15–3.33).

When examining school-level variables, about 90% of participating schools were elementary and on average served about 642 students (Table 2). Across the sample, schools had an average of 46% of economically disadvantaged students, 22% English language learners, and relatively similar percentages of white (34%), Hispanic (34%) and black/other race (32%). Schools were providing about six programs and six activities that were promoting physical activity throughout the school year. Additional descriptive information about the schools are listed in Table 2.

**Table 2:** Descriptive Information for Healthy Zone Schools, 2017–2018 School Year (Total sample = 67 schools)

Variable	n (%)	Mean (SD)
School Type		
Elementary	60 (89.6)	
Non-Elementary	7 (10.4)	
Average total number of students		642.2 (259.6)
Percent economically disadvantaged		45.6 (32.6)
Percent English Language Learnings		22.2 (20.7)
Percent Black		16.2 (12.9)
Percent Hispanic		33.9 (23.9)
Percent White		33.6 (21.1)
Percent Other Race		16.3 (15.6)
Healthy Zone Schools Cohort		
Cohort 5 (3rd year with HZS)	26 (38.8)	
Cohort 6 (2nd year with HZS)	25 (37.3)	
Cohort 7 (1st year with HZS)	16 (23.9)	
Number of HZS Physical Activity Programs (possible range: 0–12)		6.4 (1.6)
Average Program Success Rating (0–10)		8.4 (1.0)
Number of HZS Physical Activity Activities (possible range: (0–10)		6.0 (1.9)
Average Activity Success Rating (0–10)		8.5 (1.2)

All individual level variables (except for grade) were included in multivariable models. Grade level was excluded because it was providing similar information to age. Model testing revealed gender should be entered as a random coefficient and that aside from the percentage of economically disadvantaged students variable, no other school-level variables were statistically contributing to the model. Thus, there was no evidence that other school-level variables (e.g. school type, school size, cohort, etc.) were related to meeting HFZ standards for aerobic capacity. The final main effects model included gender, age, type of aerobic capacity test, and percent economically disadvantaged students. Results indicated girls had significantly lower odds to be in the HFZ for aerobic capacity compared to boys (OR = 0.53, 95% CI = 0.46–0.62) (Table 3). In addition, there was a significant inverse association indicating that students attending schools that served a greater percentage of economically disadvantaged students had lower odds to be in the HFZ for aerobic capacity (OR = 0.99, 95% CI = 0.98–0.99) (Table 3).

**Table 3:** Main Effects Model Results

Variable	OR (95% CI)	*P*-value
Gender		
Boy (referent)	1	
Girl	0.53 (0.46–0.62)	< 0.001
Age	0.74 (0.70–0.78)	< 0.001
Aerobic Capacity Test		
20 Meter Pacer (referent)	1	
15 Meter Pacer	0.25 (0.15–0.40)	< 0.001
One-mile-run	2.2 (1.39–3.40)	0.001
Percentage of Economically Disadvantaged Students	0.99 (0.98–0.99)	< 0.001

**Fig. 1:**
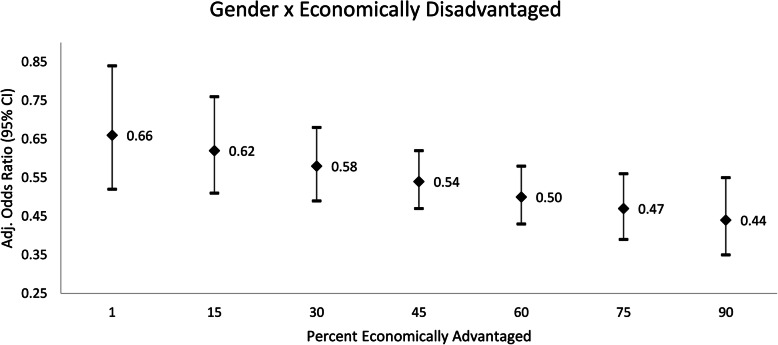
Odds Ratios for Gender across Different Levels of the Percentage of Economically Disadvantaged Students Served. Legend: Boys are the referent group

When testing the interaction between gender and the percentage of economically disadvantaged students, model results revealed a significant interaction (β = − 0.005, *p* = 0.03). This finding suggests the relation between gender and aerobic capacity was different across the percentage of economically disadvantaged students served by schools (Fig. 1). More specifically, girls (compared to boys) had even lower odds for being in the HFZ for aerobic capacity in schools serving 90% economically disadvantaged students (OR = 0.44, 95% CI = 0.35–0.55) versus schools serving 15% economically disadvantaged students (OR = 0.62, 95% CI = 0.51–0.76) (Fig. 1).

## Discussion

This study assessed the relations between gender, school SES, and cardiorespiratory fitness among elementary and middle school students. Results suggest girls in the Healthy Zone Schools have lower odds of meeting HFZ standards for aerobic capacity than boys. In addition, school-level SES was also inversely related to students’ cardiorespiratory fitness levels. Notably, students attending a school with a greater percentage of economically disadvantaged students had lower odds of meeting HFZ standards. Last, girls had lower odds of meeting HFZ standards when attending a school serving a greater percentage of economically disadvantaged students. These results highlight the importance of implementing physical activity approaches that are beneficial for boys and girls. Additionally, providing these approaches are imperative at schools serving low-income communities.

Our findings are consistent with existing literature highlighting gender and socioeconomic disparities in physical activity [2, 23]. Our study focused on an indicator of school-level SES, which may directly or indirectly contribute to lower health-related fitness levels. Schools serving low-income communities likely have additional challenges to support students’ physical activity given the existing association between SES and academic achievement [24]. For example, schools that place a lower priority on physical education are less likely to meet physical education recommendations [13]. Schools that are struggling to meet academic standards may feel the need to provide additional classroom time to catch students up, and thus may assign lower priority to physical education (or other physical activity opportunities). In our sample, the percentage of students at grade level for reading and math was highly, inversely correlated with the percentage economically disadvantaged students (− 0.93 and − 0.89, respectively). Thus, it is possible that schools serving a greater percentage of economically disadvantaged students placed a greater emphasis on additional classroom time at the expense of physical activity opportunities. As a result, students in low SES schools do not participate in as much structured physical activity during the school day compared to students at high SES schools, and therefore, miss opportunities to improve their cardiorespiratory fitness. This could be further impacted by a lack of access to structured activity outside of school for economically disadvantaged students, thereby making in-school physical activity that much more important.

Children growing up in low-income households are less likely to participate in organized sports [23]. In addition, children living in high-crime neighborhoods [25] or with less access to safe neighborhood parks participate in less physical activity [26]. Thus, the combination of fewer physical opportunities in school and more barriers to activity outside of school are likely key influential factors leading to lower fitness levels. Further, these influential factors may have a greater impact on girls’ physical activity and health-related fitness compared to boys.

Schools offering fewer physical activity opportunities may differentially impact girls’ cardiorespiratory fitness for multiple reasons. Even though girls are less active during physical education and recess, [5] these lost opportunities for physical activity may not be compensated for at other times throughout the day. Likewise, classroom-based programs are less likely to be used in low SES schools. Classroom-based programs have been shown to equally promote physical activity among boys and girls, [5, 27] which may be another factor contributing to gender disparities in health-related fitness at low SES schools. Because girls participate in less physical activity than boys throughout the day, lost opportunities to be physically active in and out of school likely have a greater impact on girls’ cardiorespiratory fitness. Even though children’s V0_2max_ is influenced by genetic factors, evidence suggests that exercise can improve children’s performance on endurance activities and that high-intensity exercise can increase children’s cardiorespiratory fitness [28–31].

Schools in our sample were offering multiple physical activity programs and activities through their participation in the HZS program. However, neither the number of programs/activities nor their perceived success were related to children’s cardiorespiratory fitness. This is likely because more information is needed about the impact of programs/activities offered (reach and effectiveness) as well as how well they were implemented. In addition, the program/success variables controlled for the number of programs and thus the specific type of programs offered may have been a more important factor. Last, it is unclear how the programs related to other opportunities such as physical education and recess, which would be expected to influence children’s fitness levels as well.

### Limitations

This study has limitations that must be considered. First, the sample of schools was from the HZS program and is not a representative sample, which impacts the generalizability of the study. Second, the observational study design limits conclusions about causal relations between variables. Third, data used to control for the number and success of physical activity programs/activities were from a self-report survey that was designed for program evaluation purposes. Thus, it is possible some respondents reported more programs/activities with greater success than what was actually implemented. Or, respondents may not have been aware of all the programs/opportunities that were offered at their school. As a result, the influence of school-level physical activity programs/activities may not have been fully accounted for. Additionally, there was no information about the amount of physical education or recess provided, which are both important factors to control for because they represent key opportunities for students to engage in physical activity during the school day. Fourth, at the school level, the percentage of Hispanic students was highly correlated to the percentage of economically disadvantaged students (Pearson r = 0.86) and thus is a potential confounder because it could not be controlled for. Notably, the percentage of students in other race/ethnicity categories were not as highly related to the percentage of economically disadvantaged students and were not significantly associated with cardiorespiratory fitness levels. Last, there was limited data at the student level. Specifically, data for student SES were not available for this study. Therefore, we were unable to determine how an individual’s SES may relate to health fitness within the context of school SES. Previous research indicates that girls attending low SES schools who self-described as “well off” (meaning their family’s standard of living was high) had greater odds for being obese compared to girls attending high SES schools who self-described as being “well off” [32]. These findings further suggest that school-level SES is an influential factor even when accounting for individual-level SES.

### Strengths

This study is one of the first to examine how school-level characteristics are associated with gender disparities in cardiorespiratory fitness. Additional study strengths include a unique data set that was generated from multiple data sources and a multilevel statistical approach that examined cross-level interactions. The analysis included FitnessGram data, which serves as an objective assessment of student’s cardiorespiratory fitness along with school-level factors collected from teacher surveys and the TEA website. Additionally, the analytic approach allowed us to examine how school-level characteristics may influence students’ fitness levels. This is an important topic area given the potential role schools play in improving health disparities and providing equitable access to physical activity opportunities to students.

## Conclusions

Findings from this study provide evidence that school-level SES is associated with students’ cardiorespiratory fitness levels and that it also likely contributes to gender disparities in health-related fitness. Schools are a key health promotion setting, although they also present many implementation challenges. A better understanding of how school characteristics influence health promotion efforts is critical to guide programming and resources. Our findings support the need for more physical activity approaches that are designed to meet the interests and needs of elementary and middle school girls. They also suggest that physical activity approaches targeted for girls are even more critical at schools serving low-income communities. More research is necessary to determine whether these findings are consistent across a representative sample of schools. In addition, more work is needed to understand how the different types of aerobic tests used may influence aerobic capacity scores, how programs delivered in the school setting influence children’s health-related fitness, and how different aspects of a comprehensive school-level approach (e.g., Whole-of-School approach) can improve existing health disparities in physical activity and cardiorespiratory fitness.

## Data Availability

The FitnessGram and Survey data that support the findings of this study are available from The Coooper Institute but restrictions apply to the availability of these data, which were used under license for the current study, and so are not publicly available. Data are however available from the authors upon reasonable request and with permission of The Cooper Institute. The School Characteristics data are available from the Texas Education Agency, https://tea.texas.gov/.

## Abbreviations

SES: Socioeconomic status
HZS: Healthy zone schools
V0_2max_: Maximal oxygen uptake
HFZ: Healthy fitness zone
TAPR: Texas academic performance report
TEA: Texas academic agency
